# Prevalence of osteoporosis and osteopenia in men and premenopausal women with celiac disease: a systematic review

**DOI:** 10.1186/s12937-019-0434-6

**Published:** 2019-02-07

**Authors:** Reza Ganji, Meysam Moghbeli, Ramin Sadeghi, Golnaz Bayat, Azita Ganji

**Affiliations:** 1 Department of Orthopedic surgery, North Khorasan University of Medical Sciences, Bojnurd, Iran; 2 Department of Modern Sciences and Technologies, Faculty of Medicine, Mashhad University of Medical Sciences, Mashhad, Iran; 3 Nuclear Medicine Research Center, Mashhad University of Medical Sciences, Mashhad, Iran; 4 Medical Student, Student Research Committee, Faculty of Medicine, Mashhad University of Medical Sciences, Mashhad, Iran; 5 Department of Gastroenterology and Hepatology, Faculty of Medicine, Mashhad University of Medical Sciences, Mashhad, Iran

**Keywords:** Celiac disease, Osteoporosis, Osteopenia, Bone mineral density

## Abstract

**Background:**

Celiac disease (CD) is known as a reason of metabolic osteopathy. Progression of non-invasive methods such as bone densitometry has shown that an important ratio of CD cases is faced with impaired bone mass and such cases are prone to bone fractures. Variety of low bone mineral density in CD is probably because of ignored confounding factors such as age, menopause, and drug. The aim of our study was to systematically review the osteoporosis and osteopenia incidences among premenopausal females and males with CD.

**Methods:**

This systematic review was done based on preferred reporting items for systematic reviews (PRISMA) guidelines. PubMed and Scopus and Cochran databases were searched according to the relevant medical subject headings (MeSH) of CD and bone mineral density until 2018. Prevalence of osteopenia and osteoporosis were used as effect size for meta-analysis. Cochrane Q (*p* < 0.05) and I^2^ index were presented to reveal the heterogeneity.

**Results:**

54 eligible full text reviews were included and nineteen selected for data extraction. Eleven articles didn’t have our inclusion criteria and had ignored confounding factors like age and menopause, and we excluded; data extraction was done in eight studies. A total of 563 premenopausal women and men who were from, UK, Brazil, India, Hungary, and Poland were included. The pooled prevalence of osteoporosis was 14.4% [95%CI: 9–20.5%] (Cochrane Q = 7.889, *p* = 0.96, I^2^ = 49.29%), and osteopenia was 39.6% [31.1–48.8%] (Cochrane Q = 14.24, *p* = 0.07, I^2^ = 71.92%), respectively.

**Conclusion:**

Our findings suggest that bone loss is more prevalent in celiac disease and can be associated with increased risk of fracture. However, but results are pooled prevalence and we need more case –control studies with more sample size and consideration of confounding factors.

## Background

Celiac disease (CD) is an auto immune disorder which is triggered by gluten in genetically susceptible cases. Despite the serologic tests, duodenal villous atrophy in pathology is also required for diagnosis of CD [1]. The current epidemiological studies show a remarkable increased incidence of celiac in the world [1–3]. Diarrhea, anemia, mal-absorption, and weight loss are the classic symptoms of CD. Non-classical CD is characterized by rheumatologic, hepatic, neurological, and musculoskeletal problems [4, 5]. CD is known as a cause of bone loss, mineral metabolism deterioration, and metabolic osteopathy [6, 7]. Interestingly, not only the low bone mineral density is reported in patients with classic malabsorption symptoms, but also it is shown in asymptomatic CD cases [8–10]. In a few studies classic presentation correlated more with sever bone loss (BMD) [11–13]. Studies has shown low BMD doesn’t have any correlation with Calcium, 25(OH) D3, and parathyroid hormone [12, 13]. Gluten free diet (GFD) may increases the BMD in these cases [9, 14–16]. Increase in cytokines in lamina properia and serum might have an important role in pathophysiological aspect of bone loss in CD cases [17, 18] and other autoimmune disorders also which are common in CD such as, thyroiditis and type I diabetes mellitus can predisposed them to the bone loss [19–21]. Low BMD make patients at risk of bone fracture and disability and even shorter height in CD [22].

High prevalence of low bone mineral density in CD from 40 to 70% has been reported due to ignorance of confounding factors such as age, endocrine disorder, smoking, and menopause [8, 23, 24]. Osteoporotic fractures accounts for 2.8 million disability-adjusted life years annually so real prevalence of bone loss in CD is important to predict and prevent from this possible problem. There are two systematic reviews in 2008 and 2015 about fracture risk and bone recovery in CD [10, 25] but there is no systematic review about prevalence of bone loss in CD. The present systematic review is performed to show the prevalence of osteoporosis and osteopenia in men and premenopausal CD.

## Methods

### Search strategy

Preferred reporting items for systematic reviews and (PRISMA) guidelines were considered to do this systematic review. PubMed and Scopus and Cochran were comprehensively searched to find the relevant published articles regarding prevalence of osteopenia before GFD in CD cases until Jan 2018. Search strategy was based on medical subject headings (MeSH) as follow: (celiac OR coeliac OR “gluten sensitive enteropathy” OR sprue) AND (bone mineral density OR densitometry OR metabolic bone disorder OR osteoporosis OR osteopenia) AND adult. All English, French and Spanish, Persian language and all date included in our search strategy. The inclusion criteria involved: Serologic and pathologic confirmation of CD, adult cases, BMD, cohort, cross-sectional, and case –control studies. Exclusion criteria also involved: postmenopausal women, BMD results with absolute values (g/cm^2^) or *Z* scores, and confounding factors on BMD such as steroid use and endocrine disorder. BMD is measured by dual-energy X-ray absorptiometry (DXA) which is reported as the standard diagnostic method of osteoporosis. By The World Health Organization (WHO) criteria, bone mass with T scores above – 1.0 is defined as normal, those between − 1.0 and − 2.4 as osteopenia or low bone mass, and those equal or below − 2.5 were considered as osteoporosis based on mostly lumbar spine and femoral neck.

### Data extraction and quality assessment

Data were extracted independently by two authors (A.G and R.G) from included articles according to the predefined parameters such as authors, publication year, number of cases, age, gender, and BMD. All of the extracted data were transferred into evidence tables. The quality checklist of STROBE (Strengthening the Reporting of Observational Studies in Epidemiology Statement) for conferences, abstracts, case control studies, cohort studies, and cross-sectional studies was used to evaluate the methodological quality of the eligible included studies. The applied checklist was consisted of different parts that estimate the validity and applicability of method and results of included studies.

### Statistical analysis

Studies with accurate quantitative data were selected for the meta-analysis using comprehensive meta-analysis (version.2). Prevalence of osteopenia and osteoporosis were used as effect size for meta-analysis. Random effects model was used for pooling data across the included studies. Cochrane Q (*p* < 0.05) and I^2^ index were presented to reveal the heterogeneity across the analyzed studies.

## Results

### Literature search

**Fig. 1:**
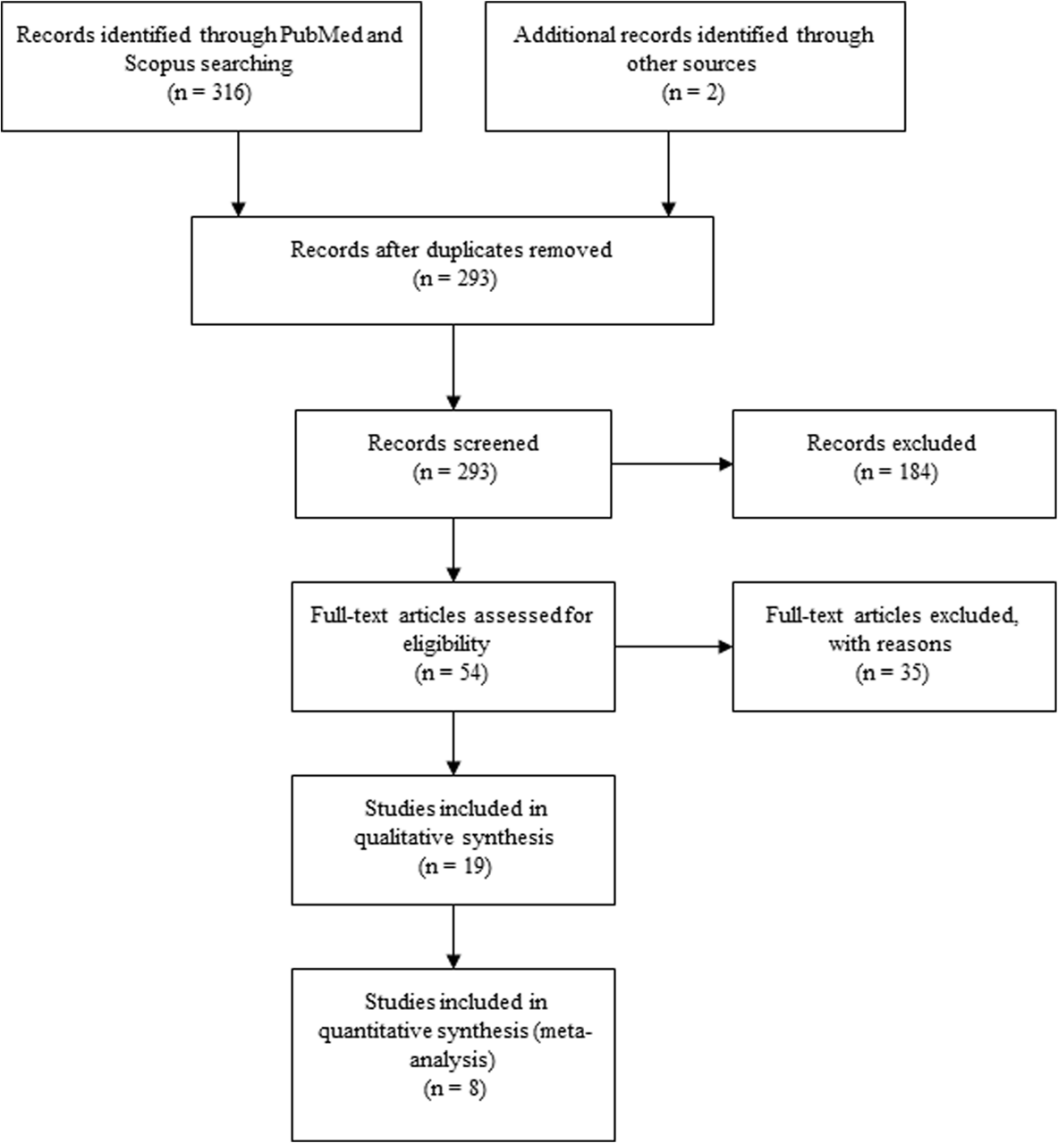
PRISMA flowchart of the study inclusion process

Totally, 396 articles were retrieved in the initial search from PubMed and Scopus. Following removing articles with duplicate citations, 342 articles were screened based on their title and abstract. Fifty-four articles were identified as the most relevant articles with the purpose of this systematic review for the full text assessment. Screening the references of the included studies and the related articles in Google scholar resulted in two more relevant studies which were included. Finally, 19 articles were eligible to be included in data extraction. Although, there was not any language restriction on our search strategy, the included articles were in English language. Eleven studies were excluded which did not have an accurate data. BMD studies with T score and separated postmenopausal women, were also included. The summary of the inclusion process of the articles is shown, according to the PRISMA flowchart (Fig. 1).

### Quality assessment and articles characteristics

Quality assessment of the included articles showed that all of the studies were cross sectional and just one of them had a control group, which reduced the quality value of most studies. Only 19 studies revealed data regarding the BMD in celiac before GFD. Eleven out of 19 reports were excluded because of the presence of menopausal women. Finally, 8 articles were acceptable and had quality for assessment. In two out of eight included studies, premenopausal and postmenopausal cases were separated and only premenopausal women or women who aged less than 55 were considered. We performed the meta-analysis in three of the included studies mentioned about femoral and lumbar densitometry separately in which two of them reported osteopenia and osteoporosis in men and women separately. Characteristics of the included studies and their results are summarized in Table 1. A total of 563 premenopausal women and men who were from UK, Brazil, India, Hungary, and Poland were included in this systematic review. The study of Duglas M et al. had the largest sample size (23 males and 105 female) [26]. We used only a part of data in Nina et al. study in premenopausal women; therefore, it had the lowest sample size (24 patients) among the eight included studies [27]. BMD in all of the studies were measured by DXA and proposed it as a diagnostic test for the low bone mass. Patients with BMD < − 1 were considered as osteopenia and BMD < − 2.5 as osteoporosis in all of the articles.

**Table 1:** Characteristics of the included studies and their results

Author, year, country	Population study	Gender (F/M)	Study design	Sample size	NBMD	osteopenia	osteoporosis
Nina, 2005, UK	< 55y/o	67%F	CS	24 out of 43	Femoral: 67% lumbar: 83%	Femoral: 29% lumbar:17%	Femoral: 4% lumbar: 0%
Kocsis, 2013, Hungary	F < 50 y/o Men⃰	73%F 32%M	CS	113	Total:46%	Total:36%	Total:18%
Singh, 2016, US	F < 50 y/o	56% F	CS	43	Total:34.9%	Total:48.8%	Total:16.3%
Sudheer, 2012, India	< 50 y/o	55%F 45%M	CS	54	Total: 39%	Total:43%	Total:18%
Pritchard, 2015, UK	< 55 y/o	66%F 33%M	CS	89	Total:56%	Total:28%	Total:5%
Meyer, 2001,US	Pre/Men**	53%F 47% M	CS	49 out of 128	Men:20% Pre:50%	Men: 80% Pre: 42%	Men: 45% Pre: %8
Szymczak, 2012, Poland	Pre /Men	83%F 17%M	CC	35	Femoral 17% Lumbar; 14%	Femoral:62/8% Lumbar: 57/2	Femoral 20% lumbar: 28/6
Silva, 2015, Brazil	< 50 y/o	No information	CS	77	Femoral:40% Lumbar:38.9%	Femoral:46.7% Lumbar:48%	Femoral:13% Lumbar:13%

Pre: premenopausal, NBMD: Normal bone mineral density

*Mean age in men was 37(18-78 yr)

**Age in men: (59 ± 15)

### Bone mineral density

**Fig. 2:**
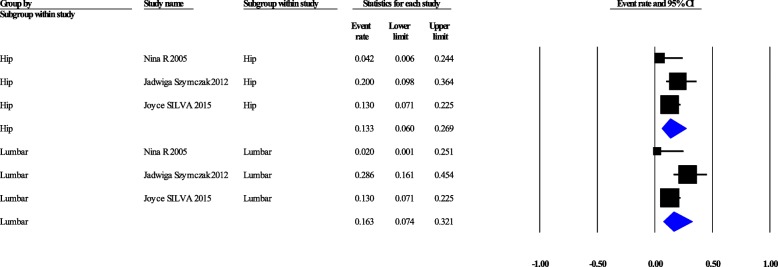
Confidence interval, osteoporosis in hip and lumbar separately

**Fig. 3:**
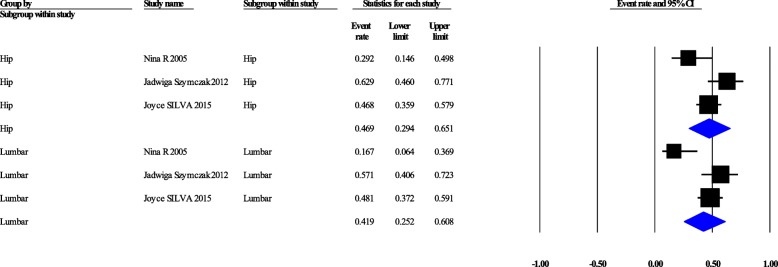
Confidence interval, osteopenia in hip and lumbar separately

**Fig. 4:**
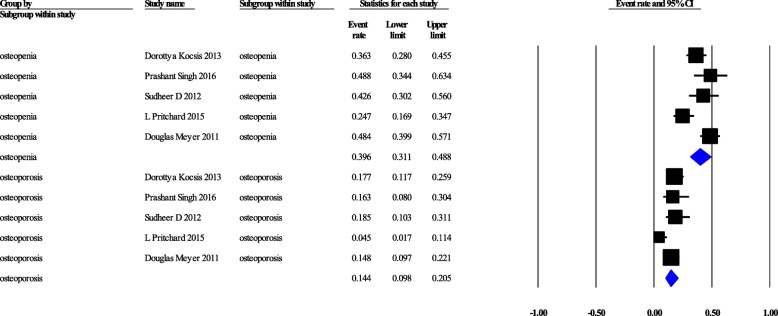
Confidence interval, overall osteopenia and osteoporosis in hip and lumbar

DXA at the femoral neck and lumbar spine are considered the gold standard to confirm the osteoporosis [28]. Moreover, DXA is one of parameters of FRAX (fracture risk assessment tool) which is a diagnostic tool for the evaluation of the 10-years probability of bone fracture risk. Pooled prevalence of osteoporosis in the hip and lumbar regions were 13.3% [95%CI: 6–26.9%] (Cochrane Q = 2.757, *p* = 0.252, I^2^ = 27.45%), and 16.3% [7.4–32.1%] (Cochrane Q = 6.62, *p* = 0.036, I^2^ = 69.82%), respectively (Fig. 2). Pooled prevalence of osteopenia in the hip and lumbar spine regions were 46.9% [29.4–65.1%] (Cochrane Q = 6.281, *p* = 0.043, I^2^ = 68.16%), and 41.9% [25.2–60.8%] (Cochrane Q = 8.839, *p* = 0.012, I^2^ = 77.37%), respectively (Fig. 3). Generally, pooled prevalence of osteopenia in lumbar and femoral was 39.6% [95%CI: 31.1–48.8%] (Q = 14.24, *P* = 0.007, I^2^ = 71.92), pooled prevalence of osteoporosis was 14.24% [95%CI: 9.8–20.5%] with (Cochrane Q = 7.88, *p* = 0.096, I^2^ = 49.29%) (Fig. 4).

## Discussion

Aim of present study was evaluation of low BMD prevalence in men and premenopausal women. Low BMD is correlated with high risk of bone fracture and results in lots of disabilities in patients. Study population in majority of publications with bone densitometry in CD included all ages and even menopausal women and in one of these case control studies 43% of cases were postmenopausal women, they didn’t separate densitometry results base on menopausal status and they didn’t mention about other confounding factors too [29]. On the other hand, we identified a few case control studies in prevalence of bone loss in CD and most of publications were cross sectional studies. In a few studies, it was shown that there was a significant difference between celiac patients and general population or control group in osteopenia and osteoporosis [11, 29]. However, Nina et al. evaluated the BMD in 43 CD patients (18–80 years old) with excluding confounding factors and they separated cases who were younger than 55 years old (24 patients) [27]. Moreover, they reported osteopenia and osteoporosis in lumbar in comparison with femoral neck. Osteoporosis in all age was 14% in spine and 7% in femur and in age less than 55 years old,femoral osteopenia in 29% and lumbar was 17% and osteoporosis in femoral was 4% with no osteoporosis in lumbar [27]. In some other reports, there wasn’t any significant difference between osteopenia and osteoporosis in femoral and spine in CD [30]. Kocsis et all evaluated BMD in 124 CD cases with excluding females who were more than 50 years old but without consideration of other confounding factors. They didn’t separate femoral from lumbar BMD, 46% of CD cases had normal BMD, 36% osteopenia, and 18% osteoporosis. Subgroup analysis showed osteoporosis in 23% and osteopenia in 36.6% of men [31]. Prashant et al. also screened the BMD in 43 patients less than 50 years old, and showed the low BMD in 65% of cases. They excluded patients with most of confounding factors include, history of vertebral fracture, chronic steroid exposure, and postmenopausal status. Prevalence of osteopenia was 48.5% and osteoporosis was 16.3% without separating the lumbar from femoral BMD [32]. An Indian report showed low BMD measurement in earlier age of CD. In this study, all CD patients were also less than 50 years old, 39% of patients had normal BMD, and 61% of newly diagnosed CD cases had low BMD and lumbar and femoral BMD were not separated, but they did not mention to the confounding risk factors for low BMD [33]. Pritcha et al. also showed that 56% of CD patients had normal BMD, 28% osteopenia, and 5% osteoporosis. They did not separate the femoral and lumbar BMD [34]. In Mayer et all study, osteoporosis and osteopenia were present in 32 to 44% of patients at the time of CD diagnosis. However, 75% of female patients were post menopause and mean age was more than 55 years old in this study. Therefore, we included 49 out of 128 patients who were men (59 ± 15 years old) and premenopausal women [26]. A polish group also evaluated the BMD in premenopausal women and men. They excluded all disease and medications which were known to affect the BMD. They had a case control study by separating densitometry of femoral from lumbar and showed significant difference in lumbar and femoral densitometry. In femoral neck, 62.8% osteopenia and 20% osteoporosis, and in lumbar spine, 57.2% osteopenia, and 28.6% osteoporosis was reported. Twenty percentages of their patients had normal BMD and there was not any comparison between male and female and BMD had negative correlation with age [35]. In Silva et al. study, whom they separated densitometry of lumar from femoral and age less than 50 years old, 40% had normal BMD in femoral and 38.9% in lumbar. Forty-six percentages of cases had femoral osteopenia and 13% had femoral osteoporosis. But in this study bone loss was more common in females in comparison with the males which was opposite of polish study. Osteopenia was observed in 41.5% of males and 75% of females even younger than 30 years old, however osteoporosis in males was more than females in this younger age goup (8.5% vs. 4.2%) but in age. 30–50 years old osteopenia and osteoporosis was more common in female and in age more than 50,Osteopenia was more common among males (66% vs. 33%), and osteoporosis was more common among females (20.6% vs. 16.6%) [36] (Table 1). Nawadays, prevalence of osteopenia and osteoporosis is more common in CD than unaffected population in the same age range [37]. In the previous studies, postmenopausal status and age has been shown as the main determinant factors of low BMD. Present systematic review, for the first time, focused on prevalence of bone loss in men and premenopausal women to exclude some confounding factors, and showed how prevalent is bone loss. This study strongly suggested that, CD should be considered as one of the leading cause of bone loss even in premenopausal women and men.

## Conclusion

There are insufficient data about osteopenia and osteoporosis in premenopausal women and men in comparison with control group. However, prevalence of osteopenia and osteoporosis are wide in different studies. It can be suggested to do more study with considering the confounding factors and sex and having control group for better risk assessment to find out best time of screening based on age and sex.

## Abbreviations

BMD: Bone mineral density
CD: Celiac disease
DXA: Dual-energy X-ray absorptiometry
GFD: Gluten free diet
MeSH: Medical subject headings
PRISMA: Preferred reporting items for systematic reviews
STROBE: Strengthening the Reporting of Observational Studies in Epidemiology Statement
WHO: World Health Organization

## References

[1] Gujral N, Freeman HJ, Thomson AB (2012). Celiac disease: prevalence, diagnosis, pathogenesis and treatment. World J Gastroenterol.

[2] Cummins AG, Roberts-Thomson IC (2009). Prevalence of celiac disease in the Asia-Pacific region. J Gastroenterol Hepatol.

[3] Rubio-Tapia A (2012). The prevalence of celiac disease in the United States. Am J Gastroenterol.

[4] Ganji A (2014). The clinical presentation of celiac disease: experiences from northeastern Iran. Middle East J Dig Dis.

[5] Hernandez L, Green PH (2006). Extraintestinal manifestations of celiac disease. Curr Gastroenterol Rep.

[6] *National Institutes of Health Consensus Development Conference Statement on Celiac Disease, June 28-30, 2004*. Gastroenterology. 2005;128(4 Suppl 1):S1–9. 10.1053/j.gastro.2005.02.007 15825115

[7] Corazza GR (2005). Bones in coeliac disease: diagnosis and treatment. Best Pract Res Clin Gastroenterol.

[8] Kalayci AG (2001). Bone mineral density and importance of a gluten-free diet in patients with celiac disease in childhood. Pediatrics.

[9] Mustalahti K (1999). Osteopenia in patients with clinically silent coeliac disease warrants screening. Lancet.

[10] Olmos M (2008). Systematic review and meta-analysis of observational studies on the prevalence of fractures in coeliac disease. Dig Liver Dis.

[11] Corazza GR (1996). Influence of pattern of clinical presentation and of gluten-free diet on bone mass and metabolism in adult coeliac disease. Bone.

[12] Corazza GR (1995). Bone mass and metabolism in patients with celiac disease. Gastroenterology.

[13] Mazure R (1994). Bone mineral affection in asymptomatic adult patients with celiac disease. Am J Gastroenterol.

[14] McFarlane XA, Bhalla AK, Robertson DA (1996). Effect of a gluten free diet on osteopenia in adults with newly diagnosed coeliac disease. Gut.

[15] Pazianas M (2005). Calcium absorption and bone mineral density in celiacs after long term treatment with gluten-free diet and adequate calcium intake. Osteoporos Int.

[16] Valdimarsson T (1996). Reversal of osteopenia with diet in adult coeliac disease. Gut.

[17] Fornari MC (1998). Pre- and post-treatment serum levels of cytokines IL-1beta, IL-6, and IL-1 receptor antagonist in celiac disease. Are they related to the associated osteopenia?. Am J Gastroenterol.

[18] Kontakou M (1995). Expression of tumour necrosis factor-alpha, interleukin-6, and interleukin-2 mRNA in the jejunum of patients with coeliac disease. Scand J Gastroenterol.

[19] Di Sabatino A, Corazza GR (2009). Coeliac disease. Lancet.

[20] Hofbauer LC (2007). Osteoporosis in patients with diabetes mellitus. J Bone Miner Res.

[21] Mosekilde L, Eriksen EF, Charles P (1990). Effects of thyroid hormones on bone and mineral metabolism. Endocrinol Metab Clin N Am.

[22] Esmaeilzadeh A (2016). Adult celiac disease: patients are shorter compared with their peers in the general population. Middle East J Dig Dis.

[23] Tau C (2006). Bone mineral density in children with celiac disease. Effect of a gluten-free diet. Eur J Clin Nutr.

[24] Vasquez H (2000). Risk of fractures in celiac disease patients: a cross-sectional, case-control study. Am J Gastroenterol.

[25] Grace-Farfaglia P (2015). Bones of contention: bone mineral density recovery in celiac disease--a systematic review. Nutrients.

[26] Meyer D (2001). Osteoporosis in a north american adult population with celiac disease. Am J Gastroenterol.

[27] Lewis NR, Scott BB (2005). Should patients with coeliac disease have their bone mineral density measured?. Eur J Gastroenterol Hepatol.

[28] *National Osteoporosis Foundation. Clinician’s guide to prevention and treatment of osteoporosis.* . 2003. Washington, DC: National Osteoporosis Foundation.

[29] Kemppainen T (1999). Osteoporosis in adult patients with celiac disease. Bone.

[30] Ganji A, Esmaeilzadeh A, Hatef M. Prevalence of Osteopenia and Osteoporosis in Patients with Celiac Disease in Northeastern Iran. Govaresh. 2012;16(4):223–7.

[31] Kocsis D (2013). Coeliac disease in a 15-year period of observation (1997 and 2011) in a Hungarian referral Centre. Eur J Intern Med.

[32] Singh P, Garber JJ (2016). Implementation and adherence to osteoporosis screening guidelines among coeliac disease patients. Dig Liver Dis.

[33] Chakravarthi SD (2012). Prevalence and predictors of abnormal bone mineral metabolism in recently diagnosed adult celiac patients. Indian J Gastroenterol.

[34] Pritchard L, Lewis SJ, Griffin J, Pearce G, Wilson S. PTU-155 Investigation of the relationship between age, gender, body mass index (BMI) and bone mineral density (BMD) as assessed by dual-energy x-ray absorptiometry (DXA) of the spine and left femur in newly diagnosed patients with coeliac disease (CD). Gut. 2015;64:A131.

[35] Szymczak J (2012). Low bone mineral density in adult patients with coeliac disease. Endokrynol Pol.

[36] Silva JT (2015). Low bone mineral density in Brazilian patients at diagnosis of celiac disease. Arq Gastroenterol.

[37] Younes M (2012). *Prevalence of bone loss in adult celiac disease and associated factors: a control case study*. Tunis Med.

